# The complete mitochondrial genome of *Dudusa sphingiformis* (Lepidoptera: Notodontidae) and phylogenetic analysis

**DOI:** 10.1080/23802359.2021.1973921

**Published:** 2021-09-17

**Authors:** Feng Zhou, Liyuan Yao, Zhibo Hou, Peng Yu, Lingyun Chen, Junyu Liang

**Affiliations:** College of Life Science, Northwest Normal University, Lanzhou, China

**Keywords:** Lepidopteran pest, mitochondrial genome, phylogenetic relationship

## Abstract

*Dudusa sphingiformis* is an important lepidopteran pest widely distributed in tropical and subtropical zones of Asia. In this paper, the complete mitochondrial genome (mitogenome) of *D. sphingiformis* was determined by next-generation sequencing. The mitogenome was 15,806 bp in length, comprising 13 protein-coding genes (PCGs), 22 tRNA genes, two rRNA genes, and an AT-rich control region (D-loop). The gene arrangement of this mitogenome was identical to that of the previous studies of Notodontidae moths. Almost all the PCGs initiated with typical ATN codons, except for *cox1* with CGA. Among them, nine PCGs terminated with TAA or TAG, while other four PCGs (*cox1*, *cox2*, *nad5*, and *nad4*) with incomplete stop codon T. All the 22 tRNAs had the typical cloverleaf structure, except for *trnS1*, whose dihydrouridine (DHU) arm forms a simple loop. Phylogenetic analysis based on the concatenated nucleotide sequences of 13 PCGs indicated that *D. sphingiformis* was more closely related to other species of family Notodontidae, forming a monophyletic group, with well-resolved relationships among five family of Noctuoidea.

Notodontidae consists of approximately 3800 known species, is a group of moths belong to superfamily Noctuoidea, which is the biggest superfamily of Lepidoptera (Miller 1992). Almost all of species Notodontidae are important injurious insects for forestry and farming (Miller 1991). To date, there have been several studies focused on the phylogenetic analysis of Notodontidae using molecular markers (Regier et al. 2017), which significantly promoted the systematic studies of Lepidoptera. The mitochondrial genome (mitogenome) provides important information for phylogenetic analysis and studies of evolutionary history (Cameron 2014). However, the complete mitogenome of the majority of species in family Notodontidae remain unsolved, expect for two species: *Clostera anachoreta* (Zhu et al. 2017) and *Clostera anastomosis* (Zhu et al. 2018). *Dudusa sphingiformis* Moore, 1872 is a member of the family Notodontidae, and widely distributed in tropical and subtropical zones of Asia (Jung and Oh 2012). This moth feeds on branches and leaves of deciduous tree, and is an important pest for agricultural industry. In the present study, we first sequenced, assembled, and annotated the complete mitochondrial genome of *D. sphingiformis* and further reconstructed the phylogenetic relationships combining with available mitogenomes of other species of Noctuoidea in GenBank.

Specimens of *D. sphingiformis* were collected in June 2020 in Huixian County of Gansu province, China (33°39′35.7″N; 106°16′11.5″E), and stored in the Institute of Zoology and Ecology, College of Life Science, Northwest Normal University, Lanzhou, China (accession number: LN2020013). The genomic DNA was extracted from muscle tissue of a single specimen’s thorax, which was sequenced by Illumina NovaSeq 6000 platform with both directions of 150 bp reads. The MitoZ v2.3 (Meng et al. 2019) was used to assemble the mitogenome based on 6 Gb clean data. The assembled mitogenome was annotated using the MITOS web server (Bernt et al. 2013) under the invertebrate mitochondrial code. The tRNA genes were confirmed by ARWEN online application (Laslett and Canback 2008). The ClustalX 2.0 software (Larkin et al. 2007) was used to align sequence dataset for phylogenetic analysis. The phylogenetic tree was built using W-IQ-TREE (Trifinopoulos et al. 2016). The newly determined genome from the present study was deposited in GenBank database (accession number: MW788876.1).

The complete mitogenome of *D. sphingiformis* was 15,806 bp in length. The A + T content of the whole genome sequence was 81.2% (40.7% A, 40.5% T, 11.7% C, and 7.1% G), indicating significant A + T bias. This mitogenome contained 13 protein-coding genes (PCGs), 22 tRNA genes, two rRNA genes, and AT-rich control region (D-loop). All of genes shared identical pattern of gene arrangement with other species of Notodontidae (Zhu et al. 2017, 2018). Like other moths of Noctuoidea (Cao et al. 2012), almost all the PCGs began with typical ATN codons (seven ATG, one ATA, and four ATT), except for *cox1* with CGA. Among them, nine PCGs terminated with TAA or TAG, while other four PCGs (*cox1*, *cox2*, *nad5*, and *nad4*) with incomplete stop codon T. All the 22 tRNAs, ranging from 65 to 72 bp, had the typical cloverleaf structure, except for *trnS1*, whose dihydrouridine (DHU) arm formed a simple loop. The absence of the DHU arm in *trnS1* was found in the mitochondrial genomes existed in most insects (Wolstenholme 1992). The two ribosomal RNA genes, *rrnL* and *rrnS,* were 1379 bp and 838 bp in length, respectively. The control regions was 297 bp long with 93.6% A + T content. This condensation or conciseness of control region was commonly demonstrated by other species of superfamily Noctuoidea (e.g. accession number: KX108766.1 and MH286069.1).

Until now, there are five species of Notodontidae, whose mitochondrial genomes with annotation have been reported. We reconstructed the phylogenetic relationships of superfamily Noctuoidea combining with available mitogenomes of other species of Noctuoidea in GenBank. These species including *D. sphingiformis* in this study belong to five family: Notodontidae, Noctuidae, Euteliidae, Nolidae, and Erebidae. We performed a maximum-likelihood (ML) analysis based on the best-fitting substitution model of GTR + F+I + G4 according to BIC with 1000 ultrafast bootstrap replicates. The concatenated nucleotide sequences of 13 PCGs from 49 species of superfamily Noctuoidea were used to construct the phylogenetic tree. Two species of family Geometridae: *Ectropis oblique* (accession number: MF417804.1) and *Biston panterinaria* (accession number: NC_020004.1) were used as outgroup for phylogenetic analyses. The results indicated that *D. sphingiformis* showed the closest phylogenetic relationship with other species of family Notodontidae, which clustered into a monophyletic group (Figure 1). The topology structure demonstrated a clear phylogenetic relationship of family Notodontidae with other populations of superfamily Noctuoidea, which were supported by the previous phylogenetic studies of Noctuoidea (Zhu et al. 2018). In this study, we characterized the complete mitochondrial genome of *D. sphingiformis* and provided new additions to studies of phylogeny construction related to Notodontidae species and other moths.

**Figure 1.**
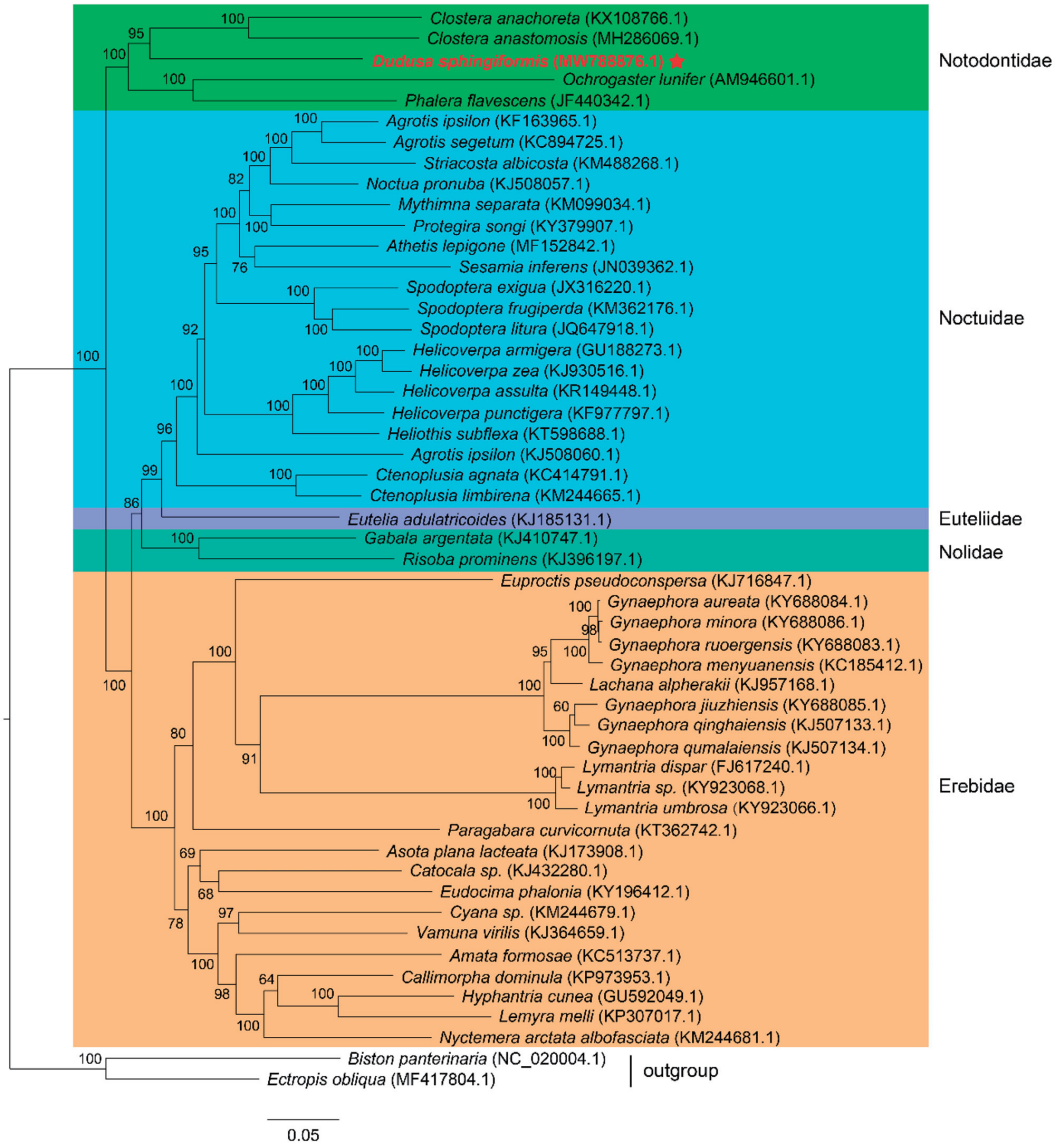
Maximum-likelihood tree showing phylogenetic relationships of *Dudusa sphingiformis* and other 49 species of superfamily Noctuoidea (including two species of superfamily Geometridae as outgroup) based on GTR + F+I + G4 model, using concatenated nucleotide sequences of 13 protein-coding genes. Numbers above or below nodes indicated the ultrafast bootstrap support values estimated with 1000 replicates. The family names and outgroup related to phylogenetic analysis were depicted at right side. The five family included Notodontidae, Noctuidae, Euteliidae, Nolidae, and Erebidae. The newly sequenced mitogenome was highlighted by the star.

## Data Availability

The genome sequence data that support the findings of this study are openly available in GenBank at https://www.ncbi.nlm.nih.gov under the accession number MW788876.1. The associated BioProject, SRA, and Bio-Sample numbers are PRJNA715946, SRR14018638, and SAMN18388692, respectively.
